# Risk Stratification of Cytogenetically Normal Acute Myeloid Leukemia With Biallelic *CEBPA* Mutations Based on a Multi-Gene Panel and Nomogram Model

**DOI:** 10.3389/fonc.2021.706935

**Published:** 2021-08-17

**Authors:** Li-Xin Wu, Hao Jiang, Ying-Jun Chang, Ya-Lan Zhou, Jing Wang, Zi-Long Wang, Lei-Ming Cao, Jin-Lan Li, Qiu-Yu Sun, Shan-Bo Cao, Feng Lou, Tao Zhou, Li-Xia Liu, Cheng-Cheng Wang, Yu Wang, Qian Jiang, Lan-Ping Xu, Xiao-Hui Zhang, Kai-Yan Liu, Xiao-Jun Huang, Guo-Rui Ruan

**Affiliations:** ^1^Beijing Key Laboratory of Hematopoietic Stem Cell Transplantation, Peking University People’s Hospital, Peking University Institute of Hematology, National Clinical Research Center for Hematologic Disease, Beijing, China; ^2^Department of Bioinformatics, AcornMed Biotechnology Co., Ltd., Beijing, China; ^3^Peking-Tsinghua Center for Life Sciences, Academy for Advanced Interdisciplinary Studies, Peking University, Beijing, China; ^4^Research Unit of Key Technique for Diagnosis and Treatments of Hematologic Malignancies, Chinese Academy of Medical Sciences, Beijing, China

**Keywords:** acute myeloid leukemia, targeted region sequencing (TRS), bi*CEBPA* mutations, risk stratification, therapy

## Abstract

**Background:**

Approximately 30% of Chinese individuals with cytogenetically normal acute myeloid leukemia (CN-AML) have biallelic *CEBPA* (bi*CEBPA*) mutations. The prognosis and optimal therapy for these patients are controversial in clinical practice.

**Methods:**

In this study, we performed targeted region sequencing of 236 genes in 158 individuals with this genotype and constructed a nomogram model based on leukemia-free survival (LFS). Patients were randomly assigned to a training cohort (*N *=111) and a validation cohort (*N* =47) at a ratio of 7:3. Risk stratification was performed by the prognostic factors to investigate the risk-adapted post-remission therapy by Kaplan–Meier method.

**Results:**

At least 1 mutated gene other than *CEBPA* was identified in patients and mutation number was associated with LFS (61.6% *vs.* 39.0%, *P* =0.033), survival (85.6% *vs.* 62.9%, *P* =0.030) and cumulative incidence of relapse (CIR) (38.4% *vs.* 59.5%, *P* =0.0496). White blood cell count, mutations in *CFS3R*, *KMT2A* and DNA methylation related genes were weighted to construct a nomogram model and differentiate two risk subgroups. Regarding LFS, low-risk patients were superior to the high-risk (89.3% *vs.* 33.8%, *P <*0.001 in training cohort; 87.5% *vs.* 18.2%, *P* =0.009 in validation cohort). Compared with chemotherapy, allogenic hematopoietic stem cell transplantation (allo-HSCT) improved 5-year LFS (89.6% *vs.* 32.6%, *P <*0.001), survival (96.9% *vs.* 63.6%, *P* =0.001) and CIR (7.2% *vs.* 65.8%, *P <*0.001) in high-risk patients but not low-risk patients (LFS, 77.4% *vs.* 88.9%, *P* =0.424; survival, 83.9% *vs.* 95.5%, *P* =0.173; CIR, 11.7% *vs.* 11.1%, *P* =0.901).

**Conclusions:**

Our study indicated that bi*CEBPA* mutant-positive CN-AML patients could be further classified into two risk subgroups by four factors and allo-HSCT should be recommended for high-risk patients as post-remission therapy. These data will help physicians refine treatment decision-making in bi*CEBPA* mutant-positive CN-AML patients.

## Introduction

Acute myeloid leukemia (AML) is one of the adult malignancies bearing the fewest mutations (1, 2). However, this disorder still comprises heterogeneous subgroups with variable responses to therapy stratified by identified leukemia driver events such as abnormalities in *FLT3*-ITD, *NPM1*, and *BCR-ABL1* fusion. Patients without adverse or favorable genetic alterations were classified into the intermediate-risk subgroup and allogenic hematopoietic stem cell transplantation (allo-HSCT) was recommended to improve survival (3). Some of the intermediate-risk patients with normal karyotype were refined as the favorable risk ones in the revised 2016 WHO classification of AML because they had the prognostically favorable alteration, biallelic *CEBPA* (bi*CEBPA*) mutations, compared with patients with wild-type or monoallelically mutated *CEBPA* (4, 5). However, this subgroup is still not homogeneous with relapse rate reaching approximately 40% (4, 6) and thus the best post-remission therapy remains controversial. Elucidation of cooperating events in this subgroup is urgently required.

Approximately 86% of AML patients have two or more driver mutations and such gene-gene interactions significantly alter the prognosis (5). To clarify the potential risk factors in bi*CEBPA* mutated AML patients, next-generation sequencing has been adopted in many studies for the detection of co-mutated genes with sensitivity reaching 1 in 10^7^ cells (7). *GATA2*, *CSF3R* and other tyrosine kinase genes (*KIT*, *JAK3* and *FLT3*-ITD), *WT1* and genes involved in chromatin/DNA modification, cohesin complex, and splicing were identified as hotspots in recent studies to decipher prognostic stratification in bi*CEBPA* mutated AML (6, 8–12). Despite promising results, the true status of these concomitant mutations and their prognostic impact on bi*CEBPA* mutated AML remain to be fully defined (13). This discordance may be attributed to two reasons. First, the sample size of bi*CEBPA* mutated AML patients was small (<100 in most studies), thus limiting the statistical significance of the conclusions to some extent. Second, dozens of genes, or just the hotspot genes, were detected, hindering analysis of the relationships among different mutations.

In addition to mutational information, clinical data are also of significance. In our previous study, we established the prognostic value of pretreatment parameter, such as higher white blood cell (WBC) count, and posttreatment parameter, such as minimal residual disease detected by multiparameter flow cytometry (MFC-MRD) in bi*CEBPA* mutated AML (14, 15). Patients with positive MFC-MRD after consolidation therapy showed a high risk of relapse and benefited from transplantation (15). Therefore, chemotherapy would no longer be appropriate as the first-line treatment for some bi*CEBPA* mutated AML patients and identification of additional risk factors is required to refine treatment decision-making. However, a comprehensive and risk-adapted estimation of the most appropriate post-remission therapy based on clinical and molecular data at diagnosis (pretreatment parameters) in this population remains to be established.

In this study, we conducted high-depth (≥1 000×) targeted region sequencing (TRS) in a large panel with 236 known and potential driver genes to investigate the mutational context in 158 newly diagnosed patients with cytogenetically normal AML (CN-AML) and bi*CEBPA* mutations. Mutational and clinical data at diagnosis were combined and weighted in a nomogram model for refined risk stratification. This study will provide practical prognosis information for bi*CEBPA* mutated CN-AML patients and pave the way for precision treatment.

## Patients and Methods

### Patients

**Figure 1:**
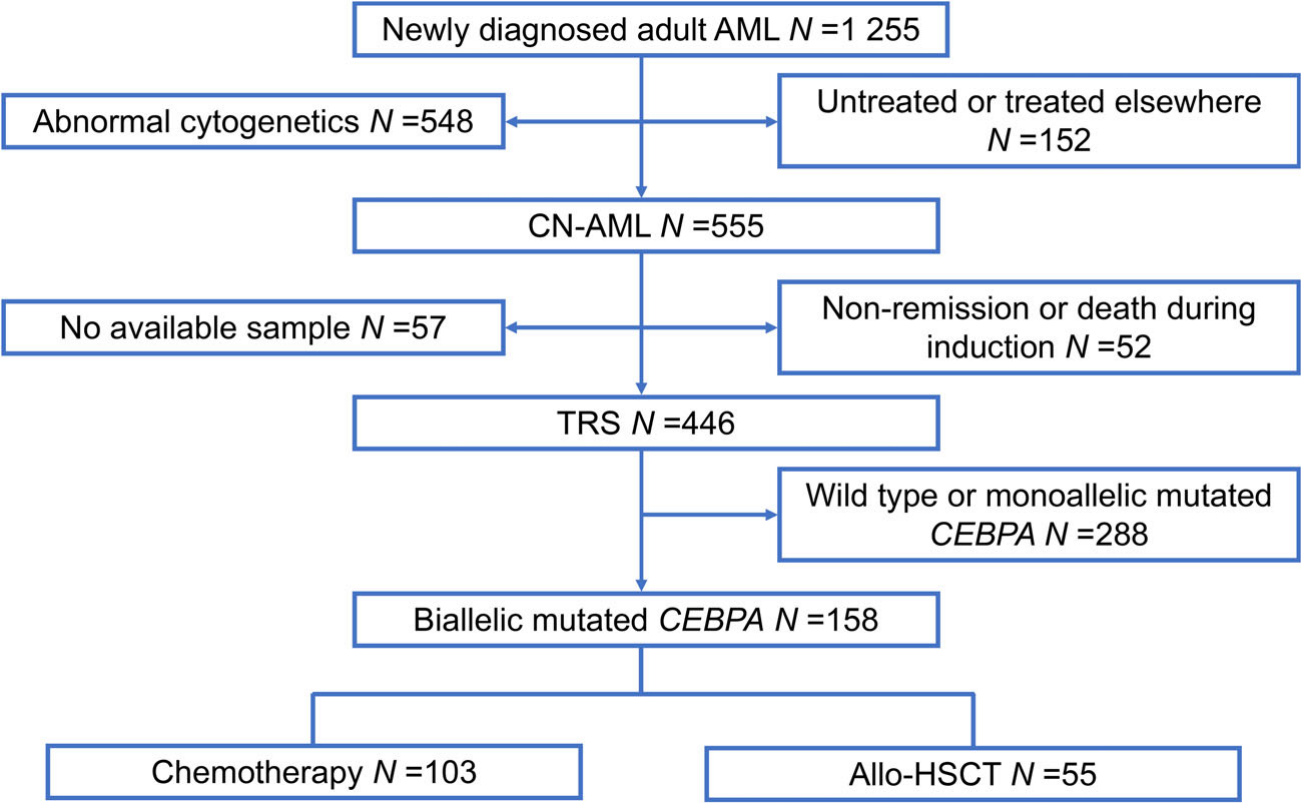
Patient recruitment and cohort assignment. AML, acute myeloid leukemia; CN, cytogenetically normal; TRS, targeted region sequencing; Allo-HSCT, allogenic hematopoietic stem cell transplantation.

A total of 1 255 patients with newly diagnosed AML were enrolled from February 2010 to December 2019 at Peking University People’s Hospital. All participants included in our study met the following criteria: (1) age ≥15 years; (2) normal cytogenetics; (3) achieved complete remission (CR); (4) bi*CEBPA* mutant-positive (**Figure 1**). In total, 158 participants qualified for subsequent analyses. The protocols for induction therapy and post-remission therapy are described in our previous study (14, 16–18). Induction treatment included 1–2 cycles of IA10 (idarubicin 10 mg/m^2^ for 3 days and cytarabine 100 mg/m^2^ for 7 days), HAA (homoharringtonine 2 mg/m^2^ for 7 days, aclarubicin 20 mg/day for 7 days and cytarabine 100 mg/m^2^ for 7 days) or CAG (cytarabine 10 mg/m^2^ every 12 hours for 14 days, aclarubicin 20 mg/day for 4 days and granulocyte-colony stimulating factor 300μg/day for 14 days). When CR was achieved, patients were recommended to receive at least 6 cycles of consolidation chemotherapy, including 4 cycles of intermediate-dose cytarabine (2 g/m^2^ every 12 hours for 3 days) and 2 or more cycles of anthracycline (daunorubicin 45 mg/m^2^ or idarubicin 10 mg/m^2^ for 3 days or mitoxantrone 8 mg/m^2^ for 3 days) in combination with cytarabine (100 mg/m^2^ for 7 days). Patients proceeded to undergo an allo-HSCT received at least 2 cycles of consolidation chemotherapy. Donors were selected from human leukocyte antigen (HLA) matched siblings, HLA matched unrelated donors or HLA haploidentical related donors. MFC-MRD monitoring was described as previously reported (15). The sensitivity was 0.01% and any measurable level of MRD was considered positive (19). For patients with positive MRD after allo-HSCT, preemptive antileukemic chemotherapy in combination with donor lymphocyte infusion (DLI) or interferon-α was given (20). For patients with hematologic relapse, chemotherapy followed by DLI was given as the first-line strategy. And for relapse prophylaxis, only DLI was used. Details of DLI were described previously (21, 22).

### High-Depth TRS and Analysis

We designed a panel of 236 known and potential driver genes for TRS (**Supplementary Table 1**). DNA was extracted from bone marrow samples using DNAzol^®^ kits (Invitrogen, Carlsbad, CA, USA) according to the manufacturer’s instructions. The sequencing process was performed according to our previous report (23). The average sequencing depth on target *per* sample was ≥1 000×. Typical mutations in *NPM1* (type A/B/D) were validated by real-time quantitative polymerase chain reaction and atypical mutations were validated by Sanger sequencing (24). Mutations in *FLT3*-ITD were validated by Sanger sequencing.

### Nomogram Model and Risk Stratification

Participants were assigned to a training cohort (*N* =111) and a validation cohort (*N* =47) at a ratio of 7:3 randomly. A nomogram was constructed based on the variables selected from the Cox regression model. The discrimination ability of the prediction model was measured by the concordance index (C-index) and the calibration was evaluated graphically by the calibration plots. Risk stratification was performed based on the nomogram model.

### Endpoints and Statistical Analyses

The primary endpoint in this study was leukemia-free survival (LFS), which was calculated from the date of CR to relapse, death from any cause, last contact, or June 30^th^, 2020. The secondary endpoints included survival, cumulative incidence of relapse (CIR) and non-relapse mortality (NRM). Survival was calculated from the date of diagnosis to death from any cause, last contact, or June 30^th^, 2020. CIR and NRM were used in a competing risk setting and death without disease progression or relapse was treated as a competing event. Continuous variables were analyzed by Mann-Whitney *U* test. Categorized variables were analyzed by Pearson Chi-square test. Survival functions were estimated using the Kaplan-Meier method and compared by the log-rank test. Variables were selected by univariate Cox regression model and those with *P <*0.15 were subsequently enrolled in the multivariate Cox regression model. Receiving an allo-HSCT was recorded as a censored event to identify the prognostic factors before an allo-HSCT. Landmark analysis was performed to revise bias from early relapse or death when comparing the outcomes of post-remission therapies. Analyses were performed using SPSS software version 22.0 (Chicago, IL, USA), GraphPad Prism 7.04^®^ (San Diego, CA, USA) and R software version 4.0.2 (http://www.Rproject.org). *P <*0.05 was considered to indicate statistical significance.

## Results

### Patient Characteristics

Among the 158 patients with bi*CEBPA* mutations, 103 received chemotherapy only, while 55 received an allo-HSCT. Rate of patients receiving an allo-HSCT was significantly decreased after 2016 than before (22.5% *vs.* 44.8%, *P* =0.003). The median time from the first CR (CR1) to receiving an allo-HSCT was 4.77 months. According to the landmark analysis, 10 patients with LFS ≤4.77 months would be excluded from the subsequent analyses unless receiving an allo-HSCT was treated as a censored event. As shown in **Table 1**, there were no significant differences between the consolidation chemotherapy and allo-HSCT cohorts in terms of sex, WBC, hemoglobin, platelets, French-American-British (FAB) type and MRD after induction (MRDint) (all *P >*0.05). Age and CR rate after first induction were significantly greater in the consolidation chemotherapy cohort than that in the allo-HSCT cohort (age, median, 41 y *vs.* 33 y, *P <*0.001; CR rate, 92.6% *vs.* 79.6%, *P* =0.021). The allo-HSCT cohort had better 5-year LFS (84.8% *vs.* 51.2%, *P <*0.001) and 5-year survival (91.9% *vs.* 74.1%, *P* =0.018), lower 5-year CIR (9.1% *vs.* 47.7%, *P <*0.001) but comparable NRM (6.1% *vs.* 1.1%, *P* =0.125) (**Supplementary Figure 1**).

**Table 1:** Patient characteristics categorized by post-remission therapy.

Variables	Consolidation Chemotherapy (*N*=94)	Allo-HSCT (CR1) (*N*=54)	*P*-value
Sex, *N* (%)			
Male	55 (58.5)	38 (70.4)	0.151
Age, y			
Median (range)	41 (17–74)	33 (15–59)	<0.001
WBC, ×10^9^/L			
Median (range)	17.27 (1.15–315.62)	18.30 (3.52–266.00)	0.164
Hemoglobin, g/L			
Median (range)	102 (47–157)	100 (58–157)	0.707
Platelets, ×10^9^/L			
Median (range)	27 (5–184)	35 (2–172)	0.363
FAB type, *N* (%)			
M2	88 (93.6)	48 (88.9)	0.483
CR after first induction, *N* (%)			
Yes	87 (92.6)	43 (79.6)	0.021
MRDint positivity, *N*/*N* (%)			
Yes	48/88 (54.5)	35/51 (68.6)	0.103

Allo-HSCT, allogenic hematopoietic stem cell transplantation; CR, complete remission; WBC, white blood cell; FAB, French–American–British; MRDint, minimal residual disease after induction.

### Genomic Analysis of bi*CEBPA* Mutated CN-AML

**Figure 2:**
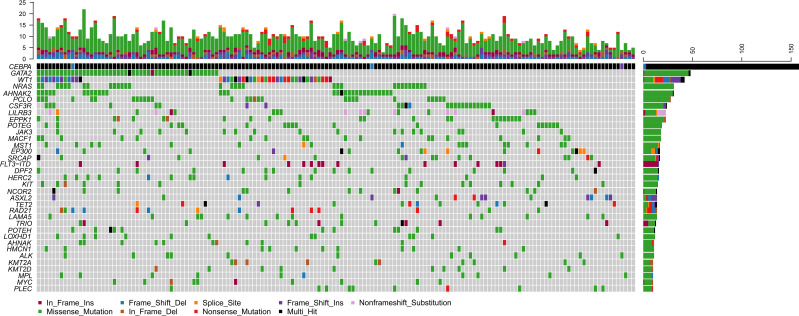
Genomic landscape of 158 CN-AML patients with bi*CEBPA* mutations. Genes mutated in ≥10 patients are shown. Boxes are colored according to the mutation type. Black box indicates multi-hit of mutation type. Non-black box of *CEBPA* indicates the same mutation type in one patient. The top bar indicates mutation load (mutation/Mb DNA) and the right bar indicates mutation frequency.

Of the 158 bi*CEBPA* mutated ones, two patients carried two frameshift deletion mutations respectively and one carried two frameshift insertion mutations (**Figure 2**). We identified 1 306 mutations in 203 genes other than *CEBPA*. The median mutation number was 8 (1–20). Interestingly, additional mutations of ≤5, 6–7, 8, 9–10, >10 were identified uniformly with ~20% of patients (**Supplementary Figure 2**). Missense mutations were the predominant type (*N* =1 024; 78.4%), followed by frame-shift (*N* =103; 7.9%), in-frame (*N* =98; 7.5%), nonsense (*N* =55; 4.2%) and splice-site (*N* =26; 2.0%) mutations. These genes (mutated in ≥10 patients) were classified into 9 genetic subgroups: transcription factors (*GATA2* and *MYC*), tumor suppressors (*WT1* and *MPL*), activated signaling (*NRAS*, *CSF3R*, *LILRB3*, *JAK3*, *MACF1*, *MST1*, *FLT3*-ITD, *KIT*, *NCOR2* and *LAMA5*), chromatin modifiers (*EP300*, *SRCAP*, *DPF2*, *ASXL2* and *ALK*), cell metabolism (*HERC2*), DNA methylation (*TET2*), cohesin complex (*RAD21*), histone methylation (*KMT2A* and *KMT2D*) and others (*AHNAK2*, *PCLO*, *EPPK1*, *POTEG*, *TRIO*, *POTEH*, *LOXHD1*, *AHNAK*, *HMCN1* and *PLEC*). Two genetic subgroups (spliceosome and adhesion) were not listed because of the low frequency of their mutated genes. *GATA2* was the most frequently affected gene in 48 patients (30.4%), followed by *WT1* (*N* =42, 26.6%), *NRAS* (*N* =31, 19.6%), *AHNAK2* (*N* =31, 19.6%), *PCLO* (*N* =28, 17.7%) and *CSF3R* (*N* =24, 15.2%). *FLT3*-ITD represented 10.1% (*N* =16) in this population and 14 of them were identified by Sanger sequencing. The missed two variants were attributed to the low mutational burden (7.7% and 7.9% respectively). Only 1 patient had *NPM1* mutation and this variant was further validated by real-time quantitative polymerase chain reaction.

**Figure 3:**
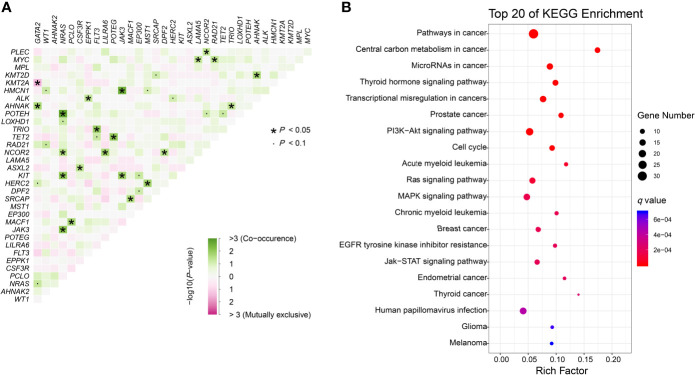
Genomic analyses. **(A)** Pairwise association between genes mutated in ≥10 patients. Green colors indicate positive association and pink colors indicate negative association. **(B)** KEGG enrichment analysis. The top 20 pathways are shown. Dot size depends on the mutation number and color depends on the *q* value (adjust *P* value).

We further identified 21 pairs of genes with co-occurrence and 1 pair with mutual exclusivity with significance (*P*<0.05, **Figure 3A**). Both *NRAS* and *NCOR2* had 4 pairwise associated genes. Mutations in *NRAS*, *JAK3* and *KIT* showed significant associations with each other. Positive pairwise associations were also found in *TET2* and *POTEG*, *GATA2* and *AHNAK*, and *CSF3R* and *ASXL2*. Only *GATA2* showed significant mutual exclusivity with *KMT2A*. KEGG pathway enrichment analysis revealed that the mutated genes, including *CEBPA*, were mainly involved in cancer (**Figure 3B**). These genes represent pan-cancer biomarkers not only in myelogenous leukemia (acute and chronic myeloid leukemia) but also in many solid tumors. Apart from several pivotal cancer-related pathways in signal transduction, we also enriched pathways in central carbon metabolism in cancer, microRNAs in cancer and EGFR tyrosine kinase inhibitor resistance.

### Mutational Context and Clinical Relevance

The general relapse rate was 32.3% (51/158) in our cohort. We further explored the correlation of mutational complexity with disease progression. A higher median mutation number was seen in ones with events (9 [2–18] *vs.* 8 [1–20]; *P* =0.050) for the patients receiving consolidation chemotherapy only. According to the median mutation number (*N* =8), patients were simply divided into two subgroups: patients with low mutational burden (mutation number <8, *N*=66) and high mutational burden (mutation number ≥8, *N* =92). Patients with low mutational burden showed significantly higher 5-year LFS (61.6% *vs.* 39.0%, *P* =0.033), higher 5-year survival (85.6% *vs.* 62.9%, *P* =0.030), lower 5-year CIR (38.4% *vs.* 59.5%, *P* =0.0496) and comparable 5-year NRM (0 *vs.* 1.5%, *P* =0.407) compared with those with high (**Supplementary Figure 3**).

### Nomogram for LFS Analysis

**Figure 4:**
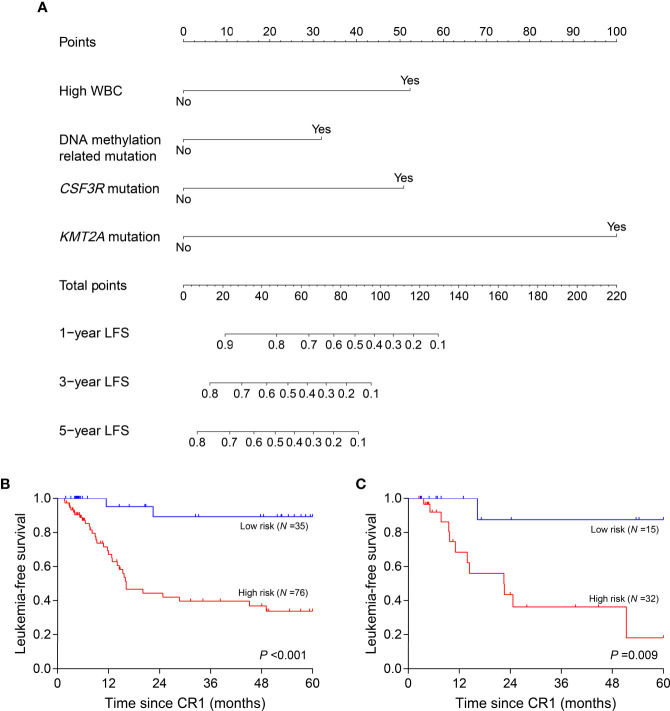
Nomogram model and risk stratification. **(A)** For WBC, “Yes” represents WBC >18.30×10^9^/L at diagnosis; for genes or genetic group, “Yes” represents mutation. **(B, C)** Leukemia-free survival analyses by risk stratification in training **(B)** and validation **(C)** cohort. WBC, white blood cell.

Clinical variables, genes, and genetic groups with mutations in ≥10 patients were enrolled for univariate analysis of LFS (allo-HSCT was recorded as a censored event). Eight variables were eligible for subsequent analysis with *P <*0.15 in the training cohort (**Supplementary Table 2**) and 4 of them eventually entered the nomogram model after multivariate Cox analysis: WBC (high *vs.* low, represents >18.30×10^9^/L *vs.* ≤18.30×10^9^/L), *CSF3R* mutation (+ *vs.* -), *KMT2A* mutation (+ *vs.* -) and DNA methylation related mutation (mutations in *TET2*, *DNMT3A*, *BAZ2A*, *IDH2* and *IDH1*, mutated in 14, 9, 5, 5 and 3 patients respectively) (+ *vs.* -) (**Figure 4A**). All points corresponding to the 4 variables were summed to predict individual probabilities of 1-, 3- and 5-year LFS. The model showed good discrimination with C-index value of 0.750 (95% confidence interval, 0.670–0.830) as well as good calibration (**Supplementary Figure 4A**). In validation cohort, the model also had good discrimination (C-index, 0.771; 95% confidence interval, 0.661–0.881) and calibration (**Supplementary Figure 4B**).

### Risk Stratification Based on Nomogram Model

According to the variables in nomogram model, patients with no identified risk factor were assigned to the low-risk subgroup (*N* =50) and the remaining to the high-risk (*N* =108). In training cohort, low-risk patients (*N* =35) showed better 5-year LFS compared with the high-risk (*N* =76, 89.3% *vs.* 33.8%, *P <*0.001) (**Figure 4B**). In the validation cohort, there were 15 patients assigned to low-risk subgroup and 32 to high-risk. The validation cohort also differentiated the two risk subgroups (low risk *vs.* high risk, 87.5% *vs.* 18.2%, *P* =0.009) (**Figure 4C**). MRDint was available in 148 patients and 59 (39.9%) ones were positive. The positive rate was significantly lower in low-risk subgroup (9/45, 20.0%) compared with high-risk (50/103, 48.5%) (*P* =0.001).

### Allo-HSCT Was Superior to Chemotherapy in High-Risk Subgroup

**Figure 5:**
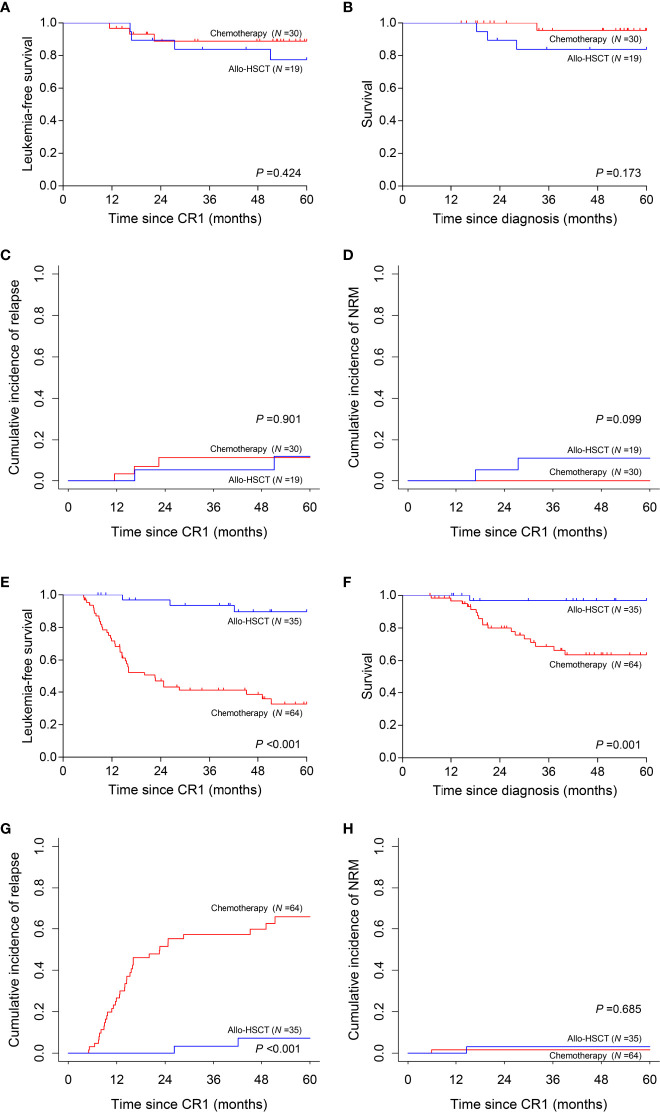
Prognosis of two post-remission therapies by risk stratification. **(A–D)** Leukemia-free survival, survival, cumulative incidence of relapse and non-relapse mortality analyses in low-risk subgroup. **(E–H)** Leukemia-free survival, survival, cumulative incidence of relapse and non-relapse mortality analyses in high-risk subgroup.

We then interrogated the effect of consolidation chemotherapy and allo-HSCT as post-remission therapies in the two risk subgroups. In the low-risk subgroup (*N* =49), there was no significant difference in the 5-year LFS (allo-HSCT *vs.* consolidation chemotherapy, 77.4% *vs.* 88.9%, *P* =0.424), 5-year survival (83.9% *vs.* 95.5%, *P* =0.173), 5-year CIR (11.7% *vs.* 11.1%, *P* =0.901) and 5-year NRM (10.9% *vs.* 0, *P* =0.099) (**Figures 5A–D**). However, in the high-risk subgroup (*N* =99), allo-HSCT was superior to consolidation chemotherapy alone (5-year LFS, 89.6% *vs.* 32.6%, *P <*0.001; 5-year survival, 96.9% *vs.* 63.6%, *P* =0.001; 5-year CIR, 7.2% *vs.* 65.8%, *P <*0.001) with comparable 5-year NRM (3.1% *vs.* 1.6%, *P* =0.685) (**Figures 5E–H**).

## Discussion

Our study presents comprehensive information on mutational context and detailed risk stratification of bi*CEBPA* mutated CN-AML patients. A significant reduction in the rate of transplantation in recent years was seen in our study. This was attributed to the important insights into bi*CEBPA* mutations in AML and recommendation for consolidation chemotherapy as the first-line post-remission therapy (25). However, in accordance with other studies (4, 6), we observed a considerable relapse rate in the CN-AML patients with bi*CEBPA* mutations. Furthermore, although not limited to CN-AML, our previous study with 36 patients identical to the current study, also supported the heterogeneity of bi*CEBPA* mutations in patients with similar relapse rate (15). It has been found that HSCT reduced the relapse rate in this population; however, the survival benefit is still controversial (26, 27). Our study indicated that allo-HSCT improved the prognosis (**Supplementary Figure 1**), demonstrating that the first-line post-remission treatment should be tailored according to an individualized risk assessment. Risk factors alone cannot represent the actual status of a patient and a comprehensive and quantitative method such as a nomogram model may provide a refined stratification. We thus sought to elucidate the heterogeneity by a large panel and develop a new prognostic model based on clinical and molecular data in this population.

As expected, higher WBC (median as the cutoff) was identified as the clinical prognostic factor. We found that at least 1 mutation cooccurred with mutated bi*CEBPA* and mutation complexity did confer higher relapse risk, which further verified the heterogeneity of bi*CEBPA* mutated CN-AML. *GATA2* mutation was the most frequent co-activated event with bi*CEBPA* mutations (6, 8, 11). Study showed that *GATA2* activity affected the mutational dynamics of leukemia in Cbfb-MYH11 knockin mice (28). The prognostic value of this gene is not well established. Several studies have revealed a trend of improvement in *GATA2* mutated CN-AML patients with bi*CEBPA* mutations (8, 29), especially when mutations disrupted the zinc finger 1 domain. In our study, *GATA2* mutation showed no correlation with prognosis (data not shown). We further identified two mutated genes (*CSF3R* and *KMT2A*) and a genetic group (DNA methylation) which conferred prognostic significance in our cohort. Braun et al. (30) confirmed that *CEBPA* mutations must be the initial event prior to mutant *CSF3R* since otherwise, AML did not develop and *CSF3R* and *CEBPA* mutations cooperated to promote leukemogenesis. *CSF3R*, which is involved in the JAK-STAT signaling pathway, is a common tyrosine kinase mutated gene in bi*CEBPA* mutated AML patients who were sensitive to JAK inhibition (9, 11, 31). The EGFR tyrosine kinase inhibitor resistance is also a pathway related to tyrosine kinase. Reports of the role of EGFR and its inhibitors (gefitinib and erlotinib) in the origination, progression and treatment of AML were discordant (32–34). Mahmud et al. (35) reported elevated protein levels of EGFR and its activation in a subset of AML and attributed the discordance in other studies to patient selection because the EGFR levels in more than 80% of AML patients did not differ from those in normal individuals. Although *EGFR* mutations were not identified in this study and its expression was not evaluated, the downstream mutated genes which were enriched in the EGFR tyrosine kinase inhibitor resistance pathway may confer drug resistance in bi*CEBPA* mutated CN-AML patients. Genes involved in DNA methylation (such as *TET2* and *DNMT3A*) were frequently mutated in bi*CEBPA* mutated AML, especially in the older participants and mutated *TET2* was not significantly different from wild type in relapse/event-free survival (6, 36). We further studied these genes as a genetic group and found that mutations in this group conferred a worse outcome. However, reports of other epigenetic modifiers involved in histone methylation are rare (13). We identified that *KMT2A*, as well as *KMT2D* and *EP300* mutations, were mutually exclusive with the most frequent *GATA2* mutation (**Figure 3A**). The infrequent mutation in KMT2 gene family members represents an obstacle to interpretation. In our study, we revealed that mutated *KMT2A* was also an independent risk factor in bi*CEBPA* mutated CN-AML patients.

Combined with sequencing data, we developed a nomogram model and further stratified the patients by the risk factors. According to our stratification, approximately one third of the patients were categorized into the low-risk subgroup, which had only bi*CEBPA* mutations and no other detrimental clinical or genetic factors. Low-risk patients were more sensitive to induction chemotherapy with lower MRD level after induction therapy. The 5-year LFS and CIR in this subgroup were not significantly improved by allo-HSCT and chemotherapy alone seemed to have better 5-year survival. That was because of the high rate of transplant-related mortality counterbalancing the graft-versus-leukemia effect in allo-HSCT. These data strongly indicated that this subgroup represented the patients with a real favorable prognosis in those with bi*CEBPA* mutated CN-AML. However, allo-HSCT was shown to be a powerful therapy to reverse the high mortality resulting from relapse in the high-risk subgroup.

One limitation of our study was the analysis of *FLT3*-ITD. The prognostic impact of *FLT3*-ITD in bi*CEBPA* mutated AML patients was controversial. Grossmann et al. (36) indicated that *FLT3*-ITD had no impact, while Zhang et al. (11) revealed that *FLT3*-ITD had worse outcome in bi*CEBPA* mutated CN-AML patients. In our study, 13 *FLT3*-ITD patients with bi*CEBPA* mutations received allo-HSCT during the CR1 (median time from CR1 to allo-HSCT, 4.53 months). The prognostic value could not be estimated because these patients were censored at the date of allo-HSCT. Although *FLT3*-ITD was more frequently observed in non-bi*CEBPA* mutated AML patients (6), the contribution of *FLT3*-ITD to risk stratification warrants further investigation because two of the *FLT3*-ITD patients receiving the consolidation chemotherapy relapsed (LFS, 20.0 months and 16.1 months respectively) eventually. Other prognostically associated genes in our study, like *CSF3R* and *KMT2A*, still need a larger and prospective study to validate.

In summary, we validated the heterogeneity of CN-AML patients with bi*CEBPA* mutations and developed a new system of risk stratification based on a nomogram model. Only one third of these patients represented the low-risk subgroup, and consolidation chemotherapy should be the first-line post-remission therapy. While in the high-risk subgroup, allo-HSCT is recommended. These data, if validated, will be greatly beneficial in translating commercial sequencing into clinical testing and directing decision-making during treatment of CN-AML patients with bi*CEBPA* mutations.

## Data Availability Statement

The sequencing data presented in the study are deposited in the NCBI Sequence Read Archive (SRA) repository, accession number PRJNA749620.

## Ethics Statement

The studies involving human participants were reviewed and approved by the Ethics Committee of Peking University People’s Hospital. Written informed consent to participate in this study was provided by the participants’ legal guardian/next of kin.

## Author Contributions

G-RR and X-JH designed the project and prepared the manuscript. L-XW, Y-LZ, Z-LW, L-MC, S-BC, FL, TZ, L-XL, and C-CW performed the experiments and statistical analyses. HJ, Y-JC, JW, J-LL, Q-YS, YW, QJ, L-PX, X-HZ, and K-YL provided the clinical data. All authors contributed to the article and approved the submitted version.

## Funding

This work was supported by grants from National Key Research and Development Program of China [Grant 2017YFA0104500], National Natural Science Foundation of China [Grant 81770156], Innovative Research Groups of the National Natural Science Foundation of China [Grant 81621001], Beijing Municipal Science and Technology Commission [Grant Z181100009618032] and Beijing Municipal Natural Science Foundation [Grant 7192213].

## Conflict of Interest

Authors S-BC, FL, TZ, L-XL, and C-CW are employed by Acornmed Biotechnology Co., Ltd.

The remaining authors declare that the research was conducted in the absence of any commercial or financial relationships that could be construed as a potential conflict of interest.

The reviewer RL declared a past co-authorship with the authors to the handling editor.

## Publisher’s Note

All claims expressed in this article are solely those of the authors and do not necessarily represent those of their affiliated organizations, or those of the publisher, the editors and the reviewers. Any product that may be evaluated in this article, or claim that may be made by its manufacturer, is not guaranteed or endorsed by the publisher.
