# Supplementary material for: Risk Stratification of Cytogenetically Normal Acute Myeloid Leukemia With Biallelic CEBPA Mutations Based on a Multi-Gene Panel and Nomogram Model
Source: Front Oncol. 2021 Aug 17;11:706935. doi: 10.3389/fonc.2021.706935 (PMC8415912; doi:10.3389/fonc.2021.706935)
Supplement: Supplementary file 1 [file DataSheet_1.docx]

**Supplementary Material**

**Supplementary Figures**


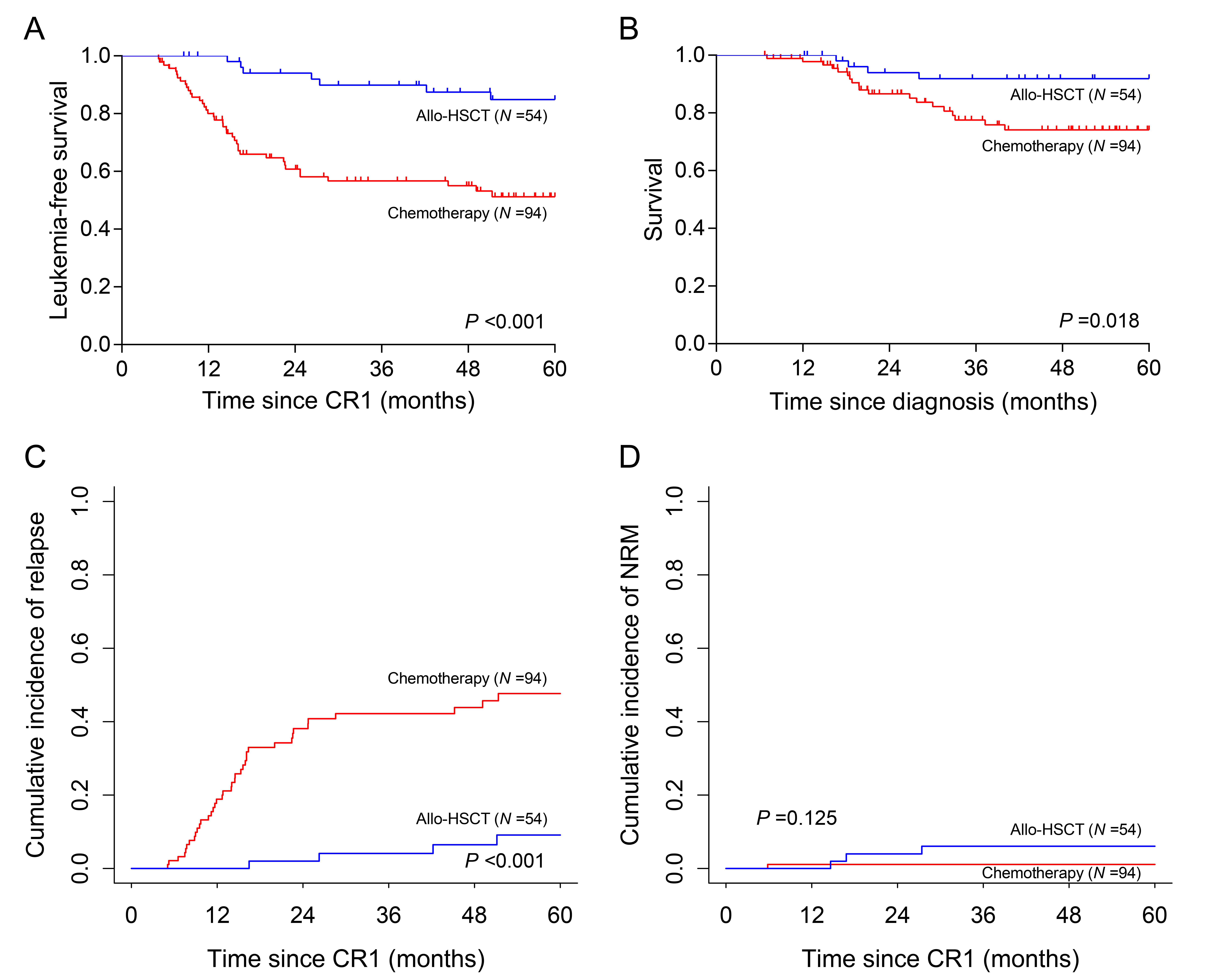


**Supplementary Figure 1. Prognosis analysis by post-remission therapies.** **(A)** Leukemia-free survival. **(B)** Survival. **(C)** Cumulative incidence of relapse. **(D)** Cumulative incidence of non-relapse mortality.

**Supplementary Figure 2. Proportion of additional mutations.** Additional mutations are classified into 5 groups with proportion of ~20% respectively.


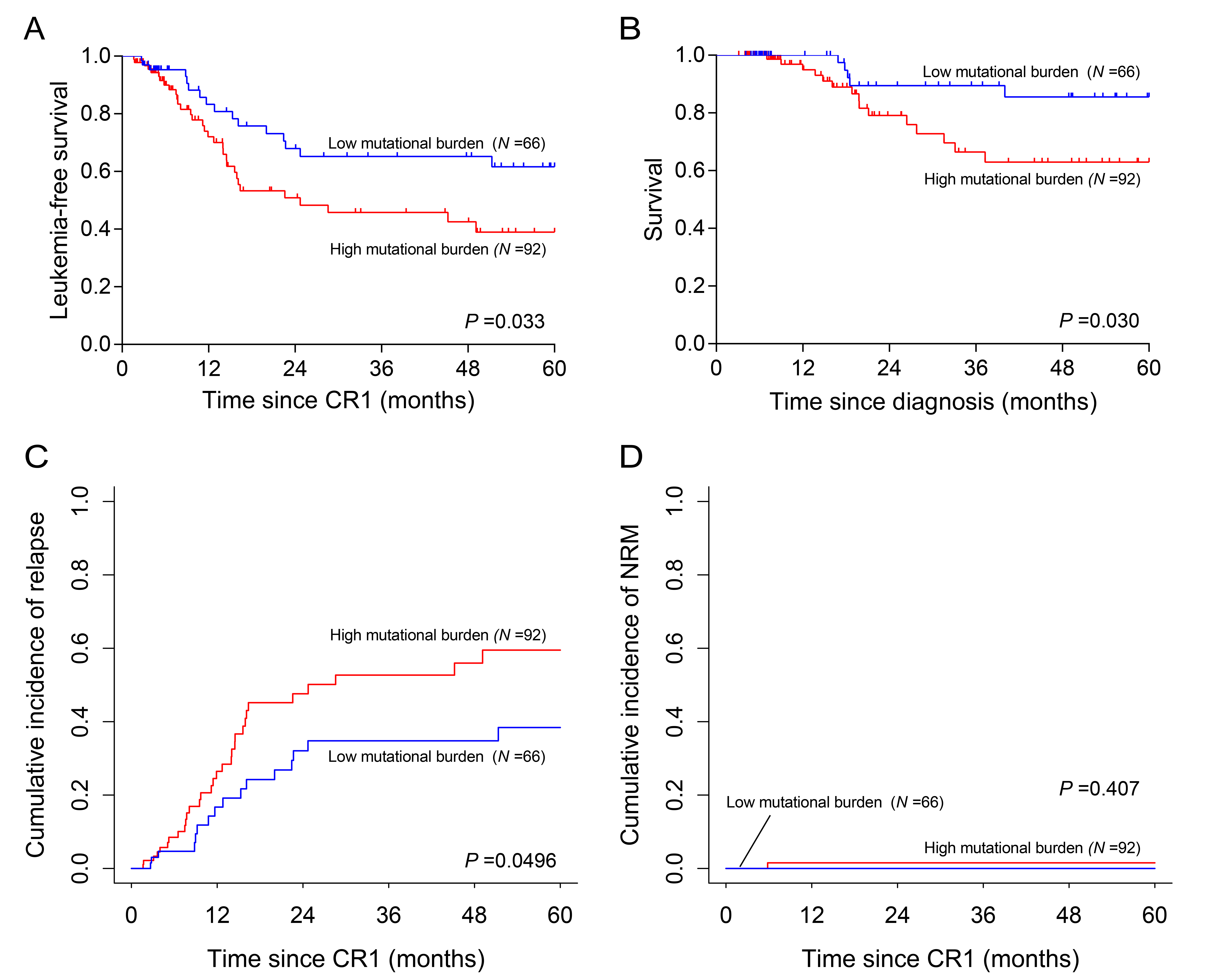


**Supplementary Figure 3. Prognosis analysis by mutational burden.** Low mutational burden represents patient with mutation number less than the median (<8) and the high represents more than the median (≥8). **(A)** Leukemia-free survival. **(B)** Survival. **(C)** Cumulative incidence of relapse. **(D)** Cumulative incidence of non-relapse mortality.


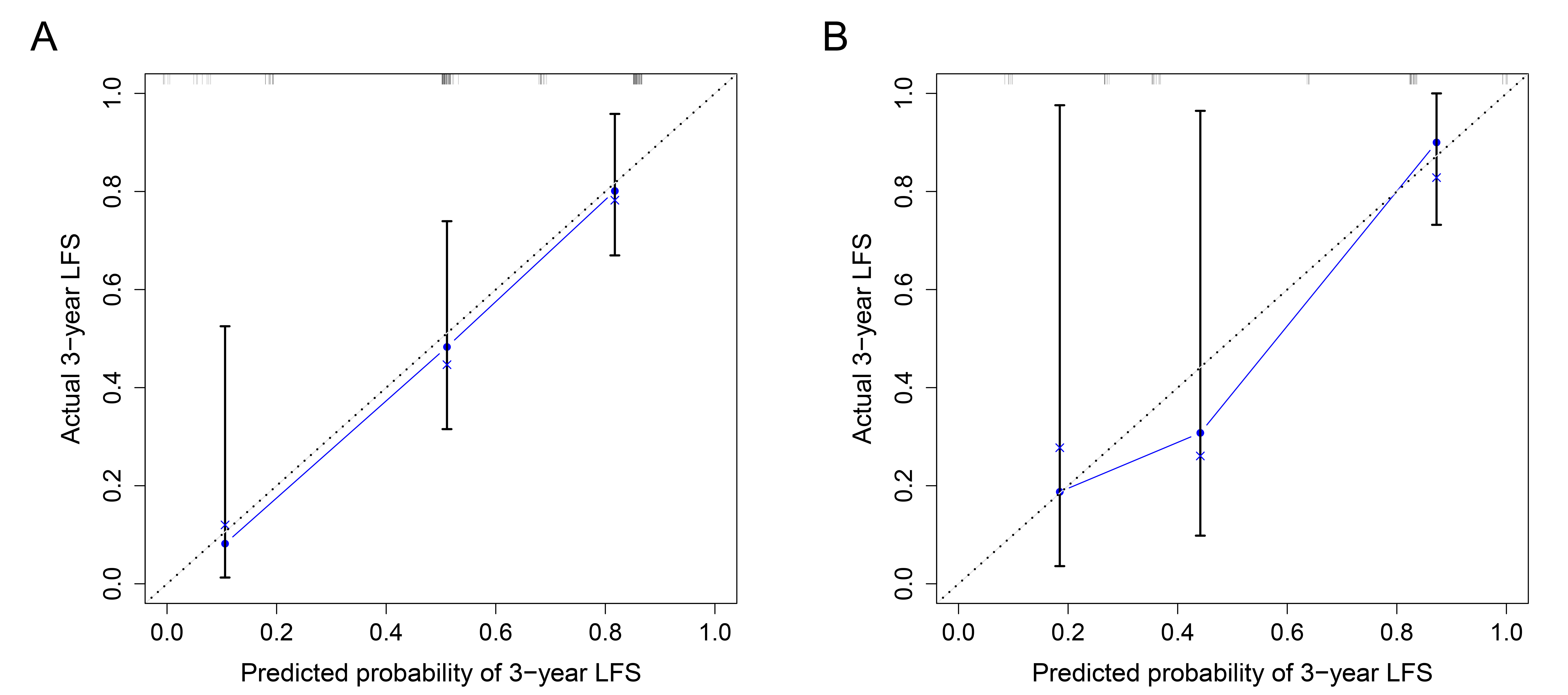


**Supplementary Figure 4. Calibration plots of the training (A) and validation (B) cohorts.** The x-axis represents the predicted 3-year LFS probabilities, and the y-axis represents the actual probabilities. The dashed line represents the ideal reference line. The blue line represents the observed results with a 95% confidence interval.

**Table**

**Supplementary Table 1. The 236-gene panel for TRS.**

| *ABCB1* | *ABCC3* | *ABCG2* | *ABL1* | *ADSL* | *AKT2* | *AKT3* |
| --- | --- | --- | --- | --- | --- | --- |
| *ALK* | *AMER1* | *ANKRD26* | *ARID1B* | *ARID2* | *ASXL1* | *ASXL2* |
| *ATM* | *ATRX* | *B2M* | *BCL11B* | *BCL2* | *BCL6* | *BCOR* |
| *BCORL1* | *BIRC3* | *BLM* | *BMP7* | *BRAF* | *BTK* | *CACNA1E* |
| *CACNA1G* | *CALR* | *CARD11* | *CBL* | *CCDC168* | *CCND1* | *CCND3* |
| *CD28* | *CD58* | *CD79A* | *CD79B* | *CDA* | *CDKN1B* | *CDKN2A* |
| *CEBPA* | *CECR2* | *CEP72* | *CHD2* | *CHD8* | *CPA2* | *CREBBP* |
| *CRLF2* | *CSF3R* | *CSMD1* | *CTCF* | *CTLA4* | *CTNNB1* | *CUX1* |
| *CXCR4* | *CYBA* | *CYP2B6* | *CYP2C19* | *CYP2C8* | *CYP3A4* | *CYP3A5* |
| *DARS* | *DCTD* | *DDX41* | *DHX15* | *DHX30* | *DIS3* | *DKC1* |
| *DNAH2* | *DNM2* | *DNMT3A* | *DOK5* | *DROSHA* | *DYNC2H1* | *EGR2* |
| *ELANE* | *EP300* | *EPOR* | *ERCC1* | *ERG* | *ETNK1* | *ETV6* |
| *EVI1* | *EZH2* | *FAM46C* | *FAT1* | *FBXW7* | *FCGR3A* | *FGFR1* |
| *FLT3* | *FOXO1* | *GATA1* | *GATA2* | *GATA3* | *GFI1* | *GNA13* |
| *GNAS* | *GSTM1* | *GSTP1* | *HAX1* | *HIST1H1E* | *HLA-DRB1* | *ID3* |
| *IDH1* | *IDH2* | *IKZF1* | *IL2RB* | *IL7R* | *IMPDH2* | *IRF4* |
| *ITPA* | *JAK1* | *JAK2* | *JAK3* | *KDM5C* | *KDM6A* | *KDM6B* |
| *KIT* | *KMT2A* | *KMT2C* | *KMT2D* | *KRAS* | *LINC00251* | *MACF1* |
| *MAP2K1* | *MAP3K7* | *MAPK1* | *MED12* | *MEF2B* | *MLH1* | *MPL* |
| *MSH6* | *MTHFR* | *MTRR* | *MYC* | *MYD88* | *NF1* | *NF2* |
| *NFATC2* | *NFKBIA* | *NFKBIE* | *NOTCH1* | *NOTCH2* | *NPM1* | *NR3C1* |
| *NRAS* | *NSD2* | *NT5C2* | *NTRK1* | *NTRK3* | *NUDT15* | *PAX5* |
| *PCLO* | *PDGFRA* | *PDGFRB* | *PHF6* | *PIGA* | *PIK3CA* | *PIK3R1* |
| *PLCG2* | *PNPLA3* | *POT1* | *PPM1D* | *PRKDC* | *PROX1-AS1* | *PRPF8* |
| *PRPS1* | *PTEN* | *PTPN11* | *RAD21* | *RB1* | *RHOA* | *RIT1* |
| *ROBO1* | *ROBO2* | *ROBO3* | *RPL10* | *RPS15* | *RRM1* | *RRM2* |
| *RRM2B* | *RUNX1* | *SAMHD1* | *SBDS* | *SERPINE1* | *SETBP1* | *SETD2* |
| *SF1* | *SF3B1* | *SH2B3* | *SLC22A1* | *SLC29A1* | *SLCO1A2* | *SLCO1B1* |
| *SMAD4* | *SMC1A* | *SMC3* | *SOCS1* | *SOD2* | *SOS1* | *SOX11* |
| *SPI1* | *SRCAP* | *SRP72* | *SRSF2* | *STAG1* | *STAG2* | *STAT3* |
| *STAT5A* | *STAT5B* | *STAT6* | *STIM1* | *SUZ12* | *TCF3* | *TERC* |
| *TERT* | *TET2* | *TNF* | *TNFAIP3* | *TNFRSF14* | *TP53* | *TPMT* |
| *TRAF3* | *TRIM24* | *U2AF1* | *UGT1A1* | *UGT1A8* | *USH2A* | *USP7* |
| *WT1* | *XPO1* | *XRCC5* | *ZMYM3* | *ZRSR2* |  |  |

**Supplementary Table 2. Univariate and Multivariate Cox analysis.**

| **Variables** | **Univariate Cox analysis** | | **Multivariate Cox analysis** | | |
| --- | --- | --- | --- | --- | --- |
|  | **HR (95% CI)** | ***P*-value** | **Coefficient** | **HR (95% CI)** | ***P*-value** |
| WBC | 3.206 (1.558, 6.597) | 0.002 | 1.487 | 4.422 (2.021, 9.674) | <0.001 |
| Hemoglobin | 1.700 (0.866, 3.334) | 0.123 |  | NS |  |
| *NRAS* | 1.926 (0.943, 3.933) | 0.072 |  | NS |  |
| *PCLO* | 0.392 (0.120, 1.280) | 0.121 |  | NS |  |
| *CSF3R* | 3.350 (1.426, 7.869) | 0.006 | 1.448 | 4.255 (1.701, 10.644) | 0.002 |
| *EPPK1* | 0.442 (0.156, 1.254) | 0.125 |  | NS |  |
| *KMT2A* | 5.915 (2.064, 16.946) | 0.001 | 2.847 | 17.242 (5.124, 58.015) | <0.001 |
| DNA methylation related gene | 1.761 (0.876, 3.541) | 0.112 | 0.906 | 2.475 (1.189, 5.152) | 0.015 |

Abbreviations: WBC, white blood cell; NS, not significant.
